# Genetically Shared Signatures Between COVID-19 and Cancer Identified Through In Silico Case–Control Analysis

**DOI:** 10.3390/genes17020150

**Published:** 2026-01-28

**Authors:** Ammar Yasir Ahmed Ahmed, Sevinç Akçay

**Affiliations:** 1 College of Pharmacy, University of Al Maarif, Al Anbar P.O. Box 55431, Iraq; ammar.yasir@uao.edu.iq; 2 Department of Molecular Biology and Genetics, Faculty of Science and Arts, Kırşehir Ahi Evran University, Kırşehir 40100, Türkiye

**Keywords:** cancer patients, breast cancer, clear cell renal cell carcinoma, triple-negative breast cancer, differentially expressed genes

## Abstract

Background/Objectives: Cancer patients are highly susceptible to infectious diseases due to malignancy- and treatment-induced immunosuppression. The coronavirus disease 2019 (COVID-19) pandemic highlighted this vulnerability, particularly in aggressive tumors such as triple-negative breast cancer (TNBC) and clear cell renal cell carcinoma (ccRCC). However, the molecular mechanisms linking cancer progression with COVID-19 severity remain poorly defined. This study aimed to identify shared molecular signatures between COVID-19 and TNBC, breast cancer, and ccRCC using integrative bioinformatics approaches. Methods: A comprehensive in silico case–control analysis was conducted using publicly available GEO transcriptomic datasets (GSE164805, GSE139038, GSE45498, and GSE105261). Differentially expressed genes (DEGs) were identified by comparing mild and severe COVID-19 cases with each cancer type. Protein–protein interaction (PPI) networks were constructed to identify hub genes, followed by Gene Ontology (GO) and Kyoto Encyclopedia of Genes and Genomes (KEGG) pathway enrichment analyses. Regulatory networks involving microRNAs (miRNAs) and transcription factors (TFs) were also examined. Results: Shared hub genes were identified across COVID-19 and cancer datasets, including *IGF1*, *MMP9*, and *NOTCH1* in TNBC; *TOP2A*, *PXN*, and *CCNB1* in breast cancer; and *ASPM* and *TTK* in ccRCC. These genes are linked to immune regulation, inflammation, cell cycle control, and tumor progression. Enrichment analyses revealed convergent pathways such as MAPK signaling, cytokine–cytokine receptor interaction, Ras signaling, and proteoglycans in cancer. Key regulatory molecules, including miR-145-5p, miR-192-5p, miR-335-5p, and transcription factors NFKB1, BRCA1, and TP53, modulated both viral and oncogenic processes. Severe COVID-19 was associated with enhanced inflammatory and proliferation-related signaling across all cancer types. Conclusions: This integrative, severity-stratified analysis identifies shared molecular and regulatory features linking severe COVID-19 with aggressive cancers, highlighting persistent immune activation and altered immune communication as common underlying themes without implying causality or clinical outcome effects. These findings provide a systems-level, hypothesis-generating framework for understanding virus–cancer interactions and may inform future biomarker discovery and immune-focused therapeutic strategies in vulnerable cancer populations.

## 1. Introduction

The coronavirus disease 2019 (COVID-19) pandemic has become a global public health challenge since its emergence in late 2019 [1]. The causative agent, severe acute respiratory syndrome coronavirus 2 (SARS-CoV-2), has been responsible for widespread morbidity and mortality worldwide [2]. Among the most vulnerable populations are individuals with cancer, who often experience more severe outcomes due to weakened immune systems resulting from the disease itself or the effects of immunosuppressive treatments such as chemotherapy and radiation. Several studies have reported increased complication and mortality rates in SARS-CoV-2-infected cancer patients [3]. Although population-level SARS-CoV-2 incidence data are available for the general population, comparative infection prevalence between healthy individuals and cancer patients remains inconsistent across studies. Current evidence indicates that cancer patients are primarily characterized by an increased risk of severe COVID-19 outcomes and mortality rather than higher infection rates [3,4].

While the acute global emergency phase of the COVID-19 pandemic has officially ended, SARS-CoV-2 continues to circulate worldwide. According to the World Health Organization (WHO) COVID-19 Dashboard, ongoing transmission persists with fluctuating regional incidence and increasingly seasonal patterns resembling those of other endemic respiratory viruses such as influenza and seasonal coronaviruses [5]. Nevertheless, COVID-19 remains a relevant public health concern, particularly for vulnerable populations including elderly and immunocompromised individuals such as cancer patients. Owing to immunosuppression associated with both malignancy and anticancer treatments, cancer patients remain at elevated risk of severe outcomes following viral infections. Importantly, immunosuppression is not uniformly present during the early stages of neoplasia and varies according to tumor type and biological context; however, early-stage tumors may already exhibit localized immune dysregulation within the tumor microenvironment, whereas systemic immunosuppression is more commonly associated with advanced disease or anticancer treatments [6]. Therefore, investigating the molecular processes shared between cancer and viral infections remains essential to inform future therapeutic strategies and improve patient outcomes during ongoing transmission and potential future outbreaks.

Around 24.2% of all new cancer cases and 15% of women’s cancer-related fatalities are caused by breast cancer, the most common cancer diagnosed in women worldwide [7]. Triple-negative breast cancer (TNBC) is an especially aggressive form of breast cancer characterized by the lack of estrogen receptor (ER), progesterone receptor (PR), and HER2/neu expression, accounting for approximately 15% of all breast cancer diagnoses [8]. TNBC is associated with high proliferative activity, early relapse, and limited targeted treatment options, resulting in a poorer prognosis compared with other breast cancer subtypes. Consequently, TNBC remains a priority for biomarker discovery and the development of novel targeted therapeutic strategies.

Clear cell renal cell carcinoma (ccRCC) is the predominant histological type of kidney cancer, accounting for approximately 80% of all renal cell carcinoma cases [9]. Significant metabolic alterations, immune evasion, and resistance to conventional therapies are characteristic of it. Like TNBC, ccRCC often exhibits an immunologically active but dysfunctional tumor microenvironment, which contributes to treatment resistance and a poor prognosis [10].

TNBC, breast cancer, and ccRCC have drawn particular attention in the context of COVID-19 due to their aggressive clinical behavior, association with immune dysfunction, and potential overlap with inflammatory and immune pathways involved in SARS-CoV-2 pathogenesis [3].

Beyond its primary respiratory manifestations, COVID-19 is increasingly recognized as a multisystem disease characterized by immune dysregulation, endothelial involvement, and systemic inflammation, features that intersect with key biological processes underlying cancer progression. Dysregulated cytokine signaling, immune suppression, and chronic inflammation—hallmarks of both cancer and severe COVID-19—may therefore contribute to worse clinical outcomes in patients with these malignancies [11,12]. Exploring shared molecular signatures between TNBC, breast cancer, ccRCC, and COVID-19 may help identify common biomarkers and therapeutic targets, ultimately informing improved clinical management of patients affected by both oncologic disease and viral infection.

Recent advances in systems and single-cell immunology have emphasized that disease severity in COVID-19 is driven not only by acute inflammation, but by persistent immune remodeling and altered immune cell communication networks, particularly in prolonged disease states such as Long COVID. A recent immune communication network analysis demonstrated that progressive COVID-19 severity is associated with declining T cell proportions, enrichment of pro-inflammatory myeloid populations, elevated cytokine signaling (including IL-32), and memory CD8^+^ T cells that exhibit increased exhaustion yet remain centrally connected within major histocompatibility complex (MHC) class I–mediated signaling networks. These findings highlight a model in which exhausted but persistently engaged immune memory cells sustain chronic inflammatory signaling through altered intercellular communication rather than through transient immune activation. Framing COVID-19 severity within this immune communication and memory-centered paradigm provides a biologically grounded context for exploring whether similar chronic immune remodeling and signaling bottlenecks are shared with aggressive cancers, where immune exhaustion, altered antigen presentation, and dysfunctional immune–tumor communication are well-established drivers of disease progression [13].

Although inflammation and immune dysregulation are widely reported in both COVID-19 and cancer, growing evidence suggests that disease severity and long-term outcomes are shaped not merely by overlapping genes, but by persistent alterations in immune communication networks and adaptive immune memory. Single-cell immune profiling has revealed that progressive COVID-19 severity is accompanied by a decline in T cell proportions, enrichment of pro-inflammatory myeloid cells, and increased exhaustion and inflammatory scores in memory CD8^+^ T cells that remain centrally connected within MHC-I–mediated communication networks, implicating sustained intercellular signaling in chronic inflammatory states such as Long COVID [13,14].

In parallel, immune exhaustion and communication imbalance are recognized features of chronic viral infection and cancer immunobiology more broadly, where deregulated antigen presentation, checkpoint molecule expression, and disrupted cytokine networks impair effective immune surveillance and memory formation [15,16].

Despite extensive research on COVID-19 and cancer individually, the shared molecular and immunological mechanisms linking SARS-CoV-2 infection with cancer-associated immune dysfunction remain incompletely understood, particularly across different cancer types and COVID-19 severity states. Many existing bioinformatics studies analyze COVID-19 without stratification by disease severity or focus on single cancer types, limiting insight into how infection intensity may differentially intersect with tumor-associated immune pathways. To address these gaps, the present study performs a severity-stratified, cross-disease in silico analysis to identify shared transcriptomic signatures between COVID-19 and three aggressive malignancies: TNBC, breast cancer, and ccRCC. By integrating differential gene expression analysis with protein–protein interaction networks, functional enrichment profiling, and transcription factor (TF)–microRNA (miRNA) regulatory network construction, this study provides a systems-level view of immune communication and regulatory convergence across severe viral infection and cancer. These findings offer biologically interpretable insights into shared pathogenic mechanisms and identify candidate biomarkers and regulatory pathways relevant to cancer patients facing ongoing and future infectious disease challenges.

## 2. Materials and Methods

### 2.1. Dataset

Gene expression profiles were retrieved from the National Center for Biotechnology Information (NCBI) Gene Expression Omnibus (GEO). Selection criteria were: (1) human tissue or whole-blood samples; (2) a case–control design; (3) no prior drug treatment; and (4) usage of a consistent microarray platform. Based on these criteria, four datasets were selected: GSE164805 (COVID-19): 5 severe, 5 mild, and 5 healthy controls. GSE164805 consisted of peripheral blood mononuclear cell (PBMC) samples collected at The First Affiliated Hospital, Zhejiang University School of Medicine between 28 January and 20 February 2020, during the early phase of the COVID-19 pandemic [17]. GSE139038 (Breast cancer): 41 tumors (24 early-stage, 17 locally advanced), 18 adjacent normals, 6 non-malignant controls [18]. GSE45498 (TNBC): 165 primary tumors, 59 adjacent normals, 54 lymph-node metastases [19]. GSE105261 (ccRCC): 9 normals, 9 primary tumors, 26 metastases [20,21]. Detailed clinicopathological information was limited or inconsistently reported across the selected GEO datasets; therefore, analyses were performed at the transcriptomic level without stratification by clinical variables.

### 2.2. Analysis of Differentially Expressed Genes and Shared Gene Signatures

Differentially expressed genes (DEGs) were identified using the GEO2R tool available through the NCBI Gene Expression Omnibus (GEO) database, which enables comparison of experimental groups using the the limma R package (version 3.54.0) implemented in R (version 4.2.2) for differential expression analysis [22,23,24]. The false discovery rate (FDR) was controlled using the Benjamini–Hochberg (BH) procedure, which adjusts raw *p*-values to account for multiple hypothesis testing while limiting the expected proportion of false positives. In this study, adjusted *p*-values (FDR) were calculated for differential expression analyses, and genes with an FDR-adjusted *p* < 0.05 were considered statistically significant. Genes with an adjusted *p*-value (FDR) < 0.05 and |log_2_ fold change (log_2_FC)| ≥ 1 were considered significantly differentially expressed, where positive and negative log_2_FC values represent upregulated and downregulated genes, respectively. The thresholds (FDR < 0.05 and |log_2_FC| ≥ 1) were selected to balance statistical stringency with biological interpretability, consistent with prior transcriptomic studies. DEGs were identified separately for the following comparisons: Mild vs. control, severe vs. control, and combined COVID-19 vs. control (GSE164805) and Breast cancer (GSE139038), TNBC (GSE45498), and ccRCC (GSE105261) vs. their respective controls. Common DEGs between COVID-19 and each cancer type were identified by generating jvenn [25]. This study did not involve any human or animal participants, as it was entirely based on publicly available datasets retrieved from the GEO database. Therefore, ethical approval and informed consent were not required.

### 2.3. Identification of Major Hub Genes from Protein Interaction Networks

Protein–protein interaction (PPI) networks were constructed utilizing the common DEGs and a high-confidence interaction score (combined score ≥ 0.7) in the Search Tool for the Retrieval of Interacting Genes/Proteins, previously Search Tool for Recurring Instances of Neighboring Genes (STRING) database (version 12.0) [26]. The Degree algorithm was selected due to its robustness and frequent use in identifying highly connected regulatory hubs in disease-associated networks. The generated networks were displayed using Cytoscape software (version 3.10.4). Hub genes were identified using the CytoHubba plugin within Cytoscape, applying the Degree algorithm to rank genes based on the number of connections in the network [27]. Hub genes are key nodes in biological networks and are often critical regulators of disease mechanisms, making them promising targets for diagnosis or therapy.

### 2.4. Biological Function Enrichment Analysis

To perform Gene Ontology (GO) functional analysis, the study used the Database for Annotation, Visualization and Integrated Discovery (DAVID) database, accessible at https://davidbioinformatics.nih.gov/tools.jsp/ (accessed on 15 July 2025). Specifically, it looked at cellular components (CC), molecular functions (MF), and biological processes (BP). The Kyoto Encyclopedia of Genes and Genomes (KEGG) pathway analysis was also incorporated into the study [28]. For each disease group (TNBC, breast cancer, and ccRCC), enrichment analyses were conducted separately in relation to mild and severe COVID-19. KEGG pathways with *p*-values less than 0.05 were regarded as significantly enriched. SRplot online tool was used to create GO and KEGG pathway enrichment graphs [29].

### 2.5. Functional Characterization of Shared Hub Genes

Following hub gene identification, shared hub genes were functionally categorized to facilitate biological interpretation of network-level findings. Classification was performed based on integration of GO biological process annotations, KEGG pathway enrichment results, and curated evidence from prior literature describing immune regulation, cell cycle control, and metabolic signaling. On this basis, hub genes were grouped into three broad functional axes: (i) immune exhaustion and chronic inflammatory signaling, (ii) antigen presentation and immune–tumor interaction, and (iii) metabolic and proliferative stress. This categorization was used as an interpretive framework to summarize and contextualize network and enrichment results, rather than as an independent statistical test.

### 2.6. Transcriptional and Post-Transcriptional Regulation of Hub Genes in COVID-19 and Breast Cancer, TNBC and ccRCC

To investigate gene regulation, the study identified TFs and miRNAs that target the hub genes. The TRRUST v2 database was used to predict TF–hub gene interactions [30] which provides curated TF–target associations based on experimental evidence. miRNA–hub gene interactions were obtained from miRDIP v4.1, which integrates data from over 30 miRNA–target prediction databases [31]. TransmiR v2.0 was utilized to generate TF–miRNA regulation networks, a comprehensive resource of TF–miRNA interactions derived from ChIP-seq and literature mining [32]. The integrated regulatory network of hub genes, TFs, and miRNAs was visualized using NetworkAnalyst.

## 3. Results

### 3.1. Shared Differentially Expressed Gene Signatures Across Disease Conditions

**Figure 1:**
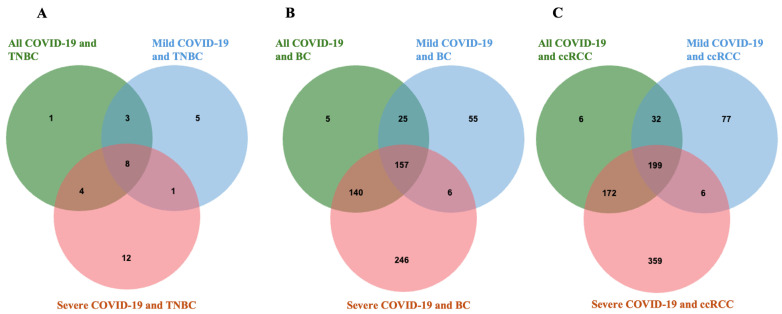
Venn diagram of common DEGs between (**A**) COVID-19 and TNBC. (**B**) COVID-19 and BC, and (**C**) COVID-19 and ccRCC. DEGs: differentially expressed genes, TNBC: triple- negative breast cancer, ccRCC: clear cell renal carcinoma, BC: breast cancer.

Venn diagram analysis revealed overlapping DEGs between COVID-19 (including mild and severe forms) and various cancer types—TNBC, BC, and ccRCC. In the COVID-19 and TNBC comparison, a total of 16 shared DEGs were identified, with 8 genes common among all COVID-19, mild COVID-19, and severe COVID-19 datasets. For BC, 157 DEGs overlapped across all three COVID-19 conditions, indicating a strong molecular relationship. In the ccRCC group, 172 DEGs were shared between all COVID-19 forms, representing the highest degree of overlap observed. Additionally, each condition displayed unique gene subsets, with mild and severe COVID-19 showing distinct DEG profiles in each cancer type (Figure 1).

### 3.2. Exploration of Protein Interaction Networks and Detection of Major Hub Genes

Cytoscape was used to visualize the PPI networks that were built using the STRING database. The top five hub genes for each condition were identified using the Degree algorithm in the CytoHubba plugin and listed in Table 1. These genes exhibit high connectivity and may play central roles in disease progression.

### 3.3. Enriched Biological Functions Associated with DEGs

**Figure 2:**
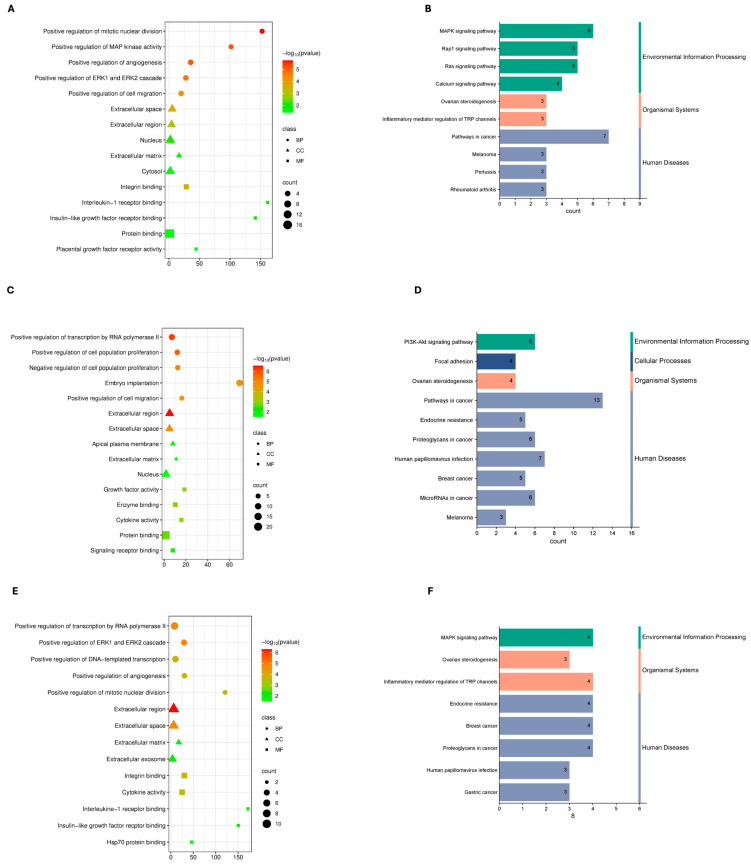
Analysis of Differential Gene Sets using Gene Ontology (GO) and KEGG Pathway Enrichment. The top enriched GO keywords for each of the three sample groups are displayed in dot plots (**A**,**C**,**E**). (**A**) TNBC and mild COVID-19, (**C**) TNBC and severe COVID-19, and (**E**) TNBC and all COVID-19. Biological Process (BP), Cellular Component (CC), and Molecular Function (MF) are the three categories into which GO words are divided. The color scale denotes statistical significance (−log_10_(*p*-value)), and the *x*-axis shows the number of genes linked to each phrase (count). Each dot’s size corresponds to the number of genes in each word. The top enriched KEGG pathways for the respective sample groups are shown in bar graphs (**B**,**D**,**F**). (**B**) TNBC and mild COVID-19, (**D**) TNBC and severe COVID-19, and (**F**) TNBC and all COVID-19. The number of genes assigned to each pathway is displayed on the *x*-axis. The bars are colored according to the KEGG categories: human diseases (blue), cellular processes (dark blue), organismal systems (orange), and environmental information processing (green).

**Figure 3:**
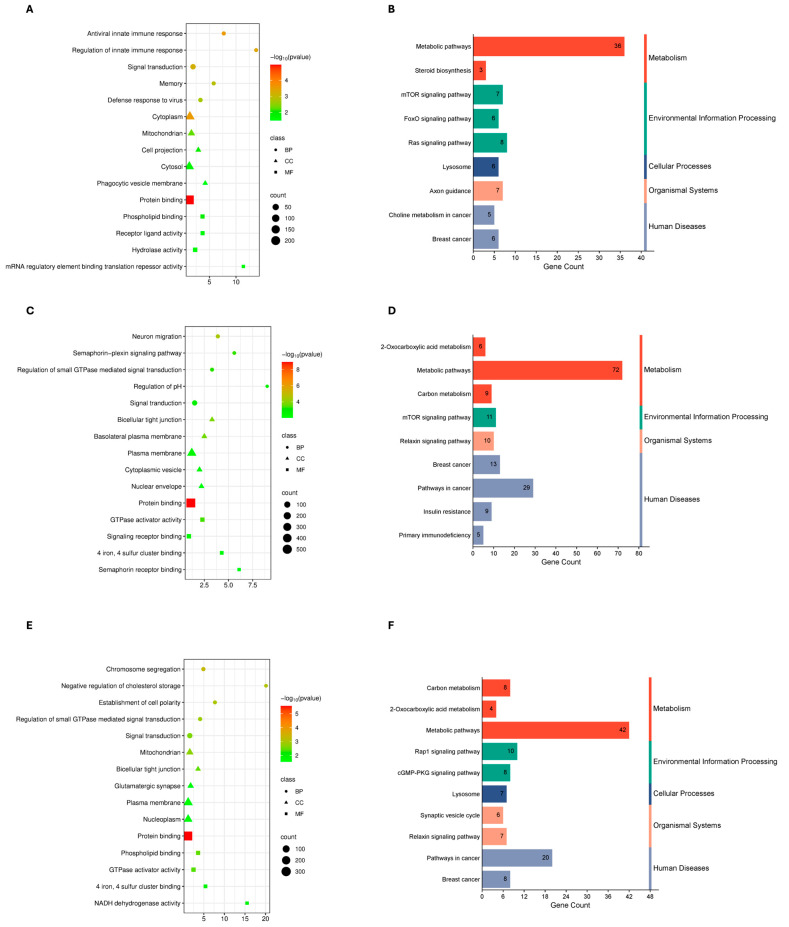
Analysis of Differential Gene Sets using Gene Ontology (GO) and KEGG Pathway Enrichment. The top enriched GO keywords for each of the three sample groups are displayed in dot plots (**A**,**C**,**E**). (**A**) ccRCC and mild COVID-19 (**C**) COVID-19 and ccRCC that is severe, (**E**) COVID-19 and ccRCC that is all. Biological Process (BP), Cellular Component (CC), and Molecular Function (MF) are the three categories into which GO words are divided. The color scale denotes statistical significance (−log_10_(*p*-value)), and the *x*-axis shows the number of genes linked to each phrase (count). Each dot’s size corresponds to the number of genes in each word. (**B**,**D**,**F**) The top enriched KEGG pathways for the respective sample groups are shown in bar charts. All COVID-19 and ccRCC, Severe COVID-19 and ccRCC, and Mild COVID-19 and ccRCC are the first three options. The number of genes assigned to each pathway is displayed on the *x*-axis. The bars are colored according to the KEGG categories: human diseases (blue), cellular processes (dark blue), organismal systems (orange), and environmental information processing (green), metabolism (red).

**Figure 4:**
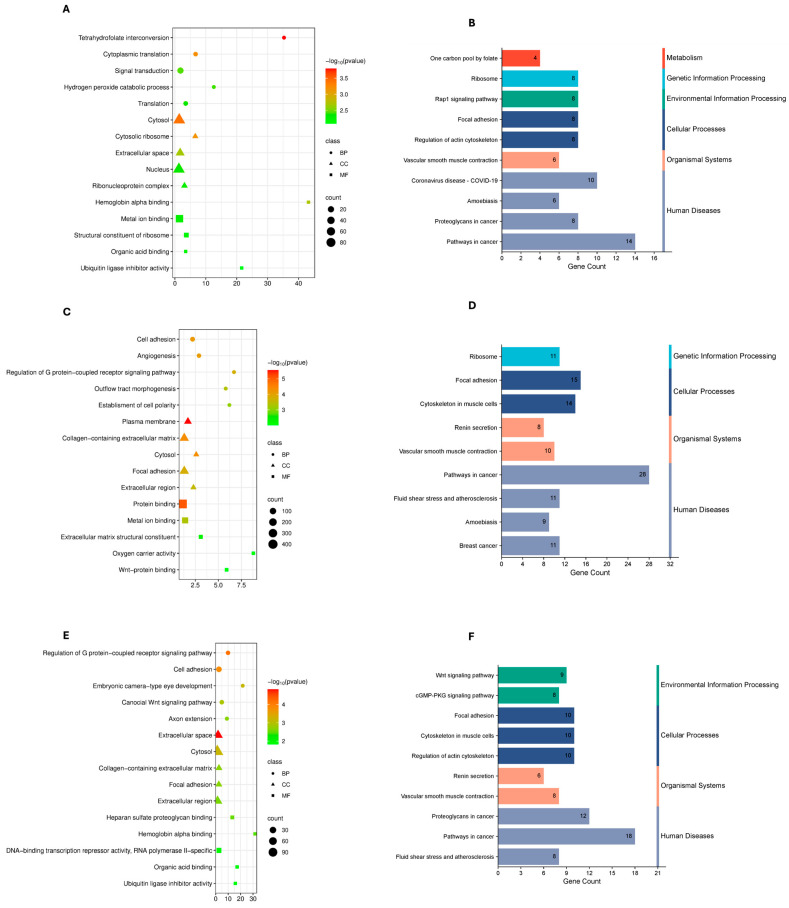
Differential gene sets are analyzed using Gene Ontology (GO) and KEGG Pathway Enrichment. Dot plots (**A**,**C**,**E**) show the top enriched GO keywords for each of the three sample groups. (**A**) mild COVID-19 and BC, (**C**) severe COVID-19 and BC, and (**E**) all COVID-19 and BC. GO terms fall into three categories: Molecular Function (MF), Cellular Component (CC), and Biological Process (BP). The *x*-axis displays the number of genes associated with each phrase (count), whereas the color scale indicates statistical significance (−log_10_(*p*-value)). The number of genes in each word is represented by the size of each dot. (**B**,**D**,**F**) Bar charts display the top enriched KEGG pathways for each sample group. (**B**) mild COVID-19 and BC; (**D**) severe COVID-19 and BC; and (**F**) COVID-19 and BC nothing. The *x*-axis shows how many genes are linked to each pathway. The bars are colored in accordance with the KEGG categories: environmental information processing (green), organismal systems (orange), cellular processes (dark blue), and human diseases (blue), metabolism (red).

To characterize the functional roles of shared DEGs, Gene Ontology and KEGG pathway enrichment analyses were performed for each cancer type under mild, severe, and combined COVID-19 conditions. Enrichment landscapes are shown in Figure 2, Figure 3 and Figure 4, and curated summaries of biologically informative categories are provided in Supplementary Tables S1–S3.

In mild COVID-19–TNBC comparisons, enriched GO biological processes included ERK/MAPK signaling, cell adhesion, and cytokine-mediated signaling, while molecular functions highlighted interleukin-1 receptor binding and integrin binding. KEGG pathways included proteoglycans in cancer and Ras signaling. In severe disease, enriched biological processes included inflammatory response, MAPK signaling, and mitotic nuclear division, with KEGG pathways such as cytokine–cytokine receptor interaction, breast cancer, and endocrine resistance. In the combined analysis, enrichment included transcriptional regulation, cell proliferation, and signal transduction, with KEGG pathways dominated by cancer-related signaling and endocrine resistance (Figure 2; Supplementary Table S1).

In COVID-19–ccRCC comparisons, enrichment was consistently dominated by cell cycle-associated processes across all severity groups. In mild cases, enriched GO terms were primarily associated with mitotic cell cycle and chromosome organization, with KEGG pathways related to cell cycle and DNA replication. In severe cases, enrichment continued to emphasize mitotic and DNA repair processes, with additional GO terms related to chemokine-associated biological processes, and KEGG pathways including cell cycle and chemokine signaling. In the combined analysis, enriched categories remained focused on chromosome organization, DNA damage response, and mitotic progression, with KEGG pathways dominated by cell cycle and p53 signaling (Figure 3; Supplementary Table S2).

For COVID-19–breast cancer comparisons, enrichment patterns differed across severity groups. In mild cases, enriched GO biological processes were dominated by DNA replication, nucleotide metabolism, and cell cycle progression, with KEGG pathways related to cell cycle and DNA replication. In severe cases, enriched categories included inflammatory response, actin cytoskeleton organization, and cell migration, with KEGG pathways such as cytokine–cytokine receptor interaction and focal adhesion. In the combined analysis, enrichment reflected both immune-related and proliferative processes, with GO terms related to immune signaling and cell proliferation, and KEGG pathways associated with cancer-related signaling and PI3K–Akt signaling (Figure 4; Supplementary Table S3).

### 3.4. Functional Enrichment Patterns of Shared Hub Genes

Functional categorization of shared hub genes demonstrated grouping into three major immune-related axes: chronic inflammatory signaling and immune exhaustion, antigen presentation and immune–tumor interaction, and metabolic–proliferative stress. Genes associated with inflammatory cytokine signaling and T cell dysfunction were predominantly enriched in the immune exhaustion axis, whereas antigen processing and presentation–related genes clustered within the immune–tumor interaction axis. A third group comprised regulators of cell cycle progression, metabolic adaptation, and proliferative signaling. *IGF1* and *ESR1* act as pleiotropic regulators bridging metabolic, proliferative, and immune-related signaling pathways and are therefore interpreted as cross-axis modulators rather than defining a separate functional category. The distribution of shared hub genes across these functional axes is summarized in Table 2.

### 3.5. Building Regulatory Networks and Determining Transcription Factors and miRNAs That Control Hub Genes

To gain insights into the regulatory mechanisms underlying the expression of hub genes shared between COVID-19 and each cancer type, TFs and miRNAs targeting the identified hub genes were predicted and integrated into regulatory networks.

**Figure 5:**
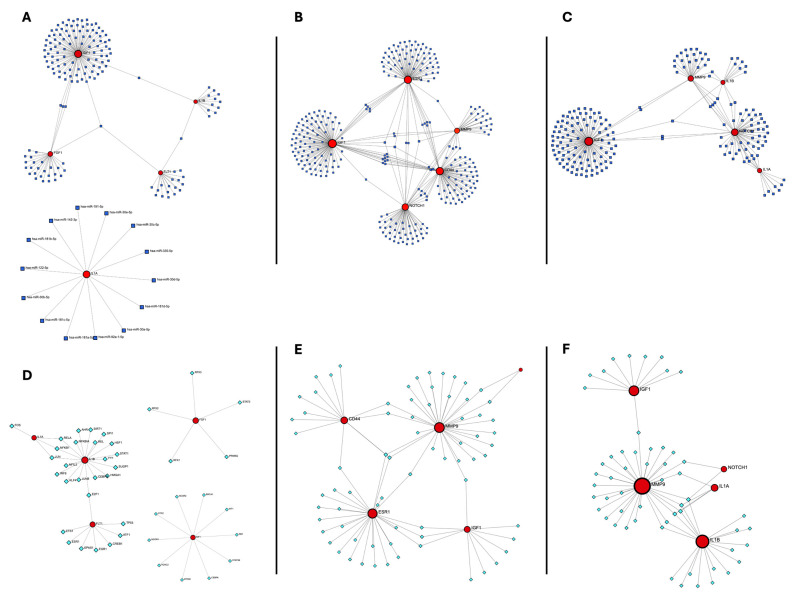
Hub gene regulatory networks in triple-negative breast cancer (TNBC) and COVID-19 (**A**–**C**). miRNA–hub gene interaction networks for (**A**) mild COVID-19 and TNBC, (**B**) severe COVID-19 and TNBC, and (**C**) all COVID-19 and TNBC cases. Circular nodes represent hub genes, and triangular nodes represent microRNAs (miRNAs). Edges indicate predicted regulatory interactions obtained from miRTarBase and integrated databases via miRDIP. (**D**–**F**) Transcription factor (TF)–hub gene regulatory networks for (**D**) mild COVID-19 and TNBC, (**E**) severe COVID-19 and TNBC, and (**F**) all COVID-19 and TNBC cases. Square nodes denote transcription factors, while circular nodes denote hub genes. Edges represent TF–target interactions derived from TRRUST v2.

In mild COVID-19–TNBC, several miRNAs were found to target at least two hub genes, including “hsa-miR-199a-3p”, “hsa-miR-24-3p”, “hsa-miR-150-3p”, “hsa-miR-130b-5p”, “hsa-miR-4753-3p”, and “hsa-miR-155-5p”. TF analysis identified E2F1, JUN, NFKB1, and RELA as regulators of at least two hub genes (Figure 5A,D). In severe COVID-19–TNBC, “hsa-miR-145-5p” targeted four hub genes, while “hsa-miR-129-5p”, “hsa-miR-9-5p”, “hsa-miR-204-5p”, and “hsa-miR-211-5p” targeted three hub genes. Additional miRNAs were identified that targeted two hub genes, as detailed in the Supplementary Tables. TF analysis showed that HDAC1 and SP1 targeted three hub genes, while several other TFs targeted two hub genes (Figure 5B,E). When all COVID-19 cases were analyzed together, “hsa-miR-24-3p” and “hsa-miR-204-5”p targeted three hub genes, and multiple miRNAs targeted two hub genes. TF analysis identified SIRT1, JUN, NFKB1, and RELA as regulators of four hub genes, with additional TFs targeting two hub genes (Figure 5C,F). Complete TF and miRNA interaction lists are provided in the Supplementary Tables S4 and S7.

**Figure 6:**
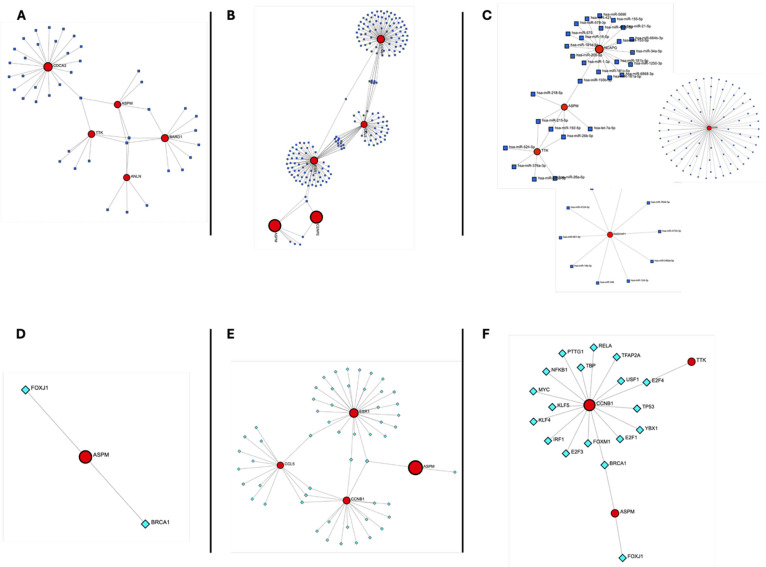
Regulatory networks of hub genes in COVID-19 and clear cell renal cell carcinoma (ccRCC). (**A**–**C**) miRNA–hub gene interaction networks for (**A**) mild COVID-19 and ccRCC, (**B**) severe COVID-19 and ccRCC, and (**C**) all COVID-19 and ccRCC cases. Circular nodes represent hub genes, and triangular nodes represent microRNAs (miRNAs). Edges indicate predicted post-transcriptional regulatory interactions sourced from miRTarBase and aggregated through miRDIP. (**D**–**F**) Transcription factor (TF)–hub gene regulatory networks for (**D**) mild COVID-19 and ccRCC, (**E**) severe COVID-19 and ccRCC, and (**F**) all COVID-19 and ccRCC cases. Square nodes represent transcription factors, and circular nodes represent hub genes. TF–target associations were curated from the TRRUST v2 database.

To characterize regulatory relationships involving hub genes shared between COVID-19 and ccRCC, miRNA–hub gene and TF–hub gene interaction networks were constructed for mild, severe, and combined COVID-19–ccRCC datasets using miRTarBase and TTRUST (Figure 6). In mild COVID-19–ccRCC, “hsa-miR-192-5p” and “hsa-miR-215-5p” each targeted four hub genes, “hsa-miR-26b-5p” targeted three hub genes, and “hsa-miR-193b-3p” targeted two hub genes. TF analysis identified FOXJ1 and BRCA1 as the only transcription factors targeting the ASPM gene in this group (Figure 6A,D). In severe COVID-19–ccRCC, hsa-miR-192-5p targeted three hub genes, while additional miRNAs targeting two hub genes were identified and are listed in the Supplementary Tables. BRCA1 targeted three hub genes, and SP1, TP53, IRF1, NFKB1, and RELA each targeted two hub genes (Figure 6B,E). When all COVID-19 cases were analyzed together, four miRNAs (“hsa-miR-193b-3p”, “hsa-miR-26b-5p”, “hsa-miR-192-5p”, and “hsa-miR-215-5p”) targeted two hub genes. BRCA1 and E2F4 were the only TFs targeting two hub genes in this combined analysis (Figure 6C,F). Complete miRNA and TF interaction data are provided in Supplementary Tables S5 and S8.

**Figure 7:**
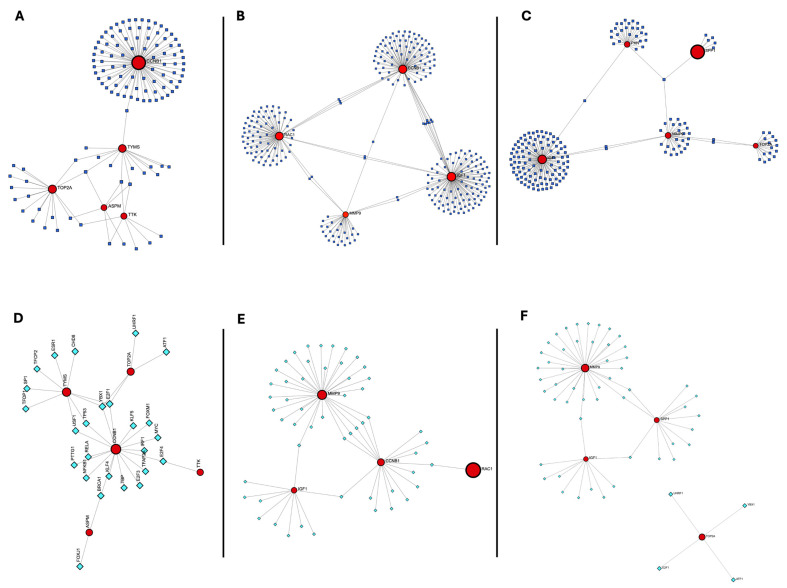
Regulatory networks of hub genes in COVID-19 and breast cancer. (**A**–**C**) miRNA–hub gene interaction networks for (**A**) mild COVID-19 and breast cancer, (**B**) severe COVID-19 and breast cancer, and (**C**) all COVID-19 and breast cancer cases. Circular nodes indicate hub genes, while triangular nodes represent microRNAs (miRNAs). Edges correspond to predicted regulatory interactions derived from miRTarBase and integrated using miRDIP. (**D**–**F**) Transcription factor (TF)–hub gene regulatory networks for (**D**) mild COVID-19 and breast cancer, (**E**) severe COVID-19 and breast cancer, and (**F**) all COVID-19 and breast cancer cases. Square nodes represent transcription factors, and circular nodes denote hub genes. TF–target gene associations were sourced from the TRRUST v2 database.

In mild COVID-19–breast cancer, three miRNAs (“hsa-miR-193b-3p”, “hsa-miR-26b-5p”, and “hsa-miR-215-5p”) targeted three hub genes, while five miRNAs targeted two hub genes (“hsa-let-7b-5p”, “hsa-miR-203a-3p”, “hsa-miR-524-5p”, “hsa-miR-218-5p”, and “hsa-miR-192-5p”). TF analysis identified E2F1 and YBX1 as regulators of three hub genes, and BRCA1, E2F4, TP53, and USF1 as regulators of two hub genes (Figure 7A,D). In severe COVID-19–breast cancer, seventeen miRNAs targeting two hub genes were identified, with complete lists provided in the Supplementary Tables. Eight TFs (EP300, BRCA1, IRF1, KLF5, NFKB1, RELA, TFAP2A, and PTTG1) targeted two hub genes (Figure 7B,E). When all COVID-19 cases were combined, hsa-miR-335-5p targeted three hub genes, while five miRNAs targeted two hub genes (“hsa-miR-145-5p”, “hsa-let-7e-5p”, “hsa-miR-302a-5p”, “hsa-miR-21-5p”, and “hsa-miR-524-5p”). TF analysis identified EP300, HDAC1, SP1, and CEBPA as regulators of two hub genes (Figure 7C,F). Complete miRNA and TF interaction data are provided in the Supplementary Tables S6 and S9.

Table 3 integrates severity-stratified molecular features across TNBC, breast cancer, and ccRCC, revealing a shared progression from early immune activation in mild COVID-19 to persistent immune communication dysfunction, metabolic stress, and immune exhaustion in severe disease.

## 4. Discussion

This study presents a comprehensive, severity-stratified in silico analysis of shared molecular features between COVID-19 and three aggressive cancer types—TNBC, breast cancer, and ccRCC. By integrating transcriptomic differential expression analysis with PPI networks, functional enrichment profiling, and TF–miRNA regulatory network construction, we systematically characterized genes, pathways, and regulatory elements commonly observed across SARS-CoV-2 infection and tumor contexts. In contrast to prior bioinformatics studies that examined COVID-19 or cancer in isolation or without stratification by infection severity, our analyses indicate that increasing COVID-19 severity is accompanied by a progressive enrichment of oncogenic, inflammatory, and immune-related signaling signatures across all three cancer types. These observations support the presence of a shared, severity-dependent molecular convergence in which severe viral infection coincides with pre-existing immune dysregulation and signaling vulnerabilities characteristic of aggressive cancers. Collectively, this underscores the importance of accounting for infection severity when interpreting virus–cancer molecular interactions, while remaining hypothesis-generating and not implying direct clinical or causal effects.

Across all analyses, increasing COVID-19 severity was consistently associated with progressive enrichment of immune-related, inflammatory, and proliferative signaling programs shared with aggressive cancers. Rather than reflecting random overlap, this pattern suggests that severe SARS-CoV-2 infection and aggressive tumors engage partially overlapping molecular programs related to immune stress, metabolic adaptation, and intercellular communication. These findings support a model of severity-dependent molecular convergence, in which intense viral immune activation coincides with signaling vulnerabilities that are already characteristic of aggressive tumor microenvironments.

In the COVID-19–TNBC comparison, recurrent hub genes (*NOTCH1*, *IGF1*, *MMP9*, *IL1A*, *IL1B*) were functionally linked to immune modulation, extracellular matrix remodeling, inflammatory signaling, and cell–cell communication [38]. Rather than representing isolated inflammatory markers, these genes converged on pathways governing immune–tumor interaction and chronic immune activation. Notably, *IGF1* was consistently identified across mild and severe COVID-19–TNBC analyses, suggesting a sustained role in integrating infection-driven immune responses with TNBC-associated metabolic and proliferative signaling [39]. This persistence across severity strata supports involvement in longer-term immune remodeling rather than transient inflammatory responses alone.

In ccRCC, shared hub genes were dominated by regulators of cell-cycle progression and mitotic control (*ASPM*, *CCNB1*, *TTK*, *RAD51AP1*, *NCAPG*), reflecting proliferative and metabolic stress within an immune-inflamed yet functionally constrained tumor microenvironment. The consistent identification of *ASPM* across severity groups suggests stable coupling between proliferative programs and immune dysregulation under systemic inflammatory stress. These findings align with the known biology of ccRCC, where immune infiltration coexists with immune exhaustion and aberrant angiogenic and metabolic signaling [40].

In breast cancer excluding TNBC, influential hubs (*SPP1*, *TOP2A*, *IGF1*, *MMP9*, *PXN*) highlighted integration of immune signaling, extracellular matrix dynamics, and DNA replication processes. Severity-dependent shifts were observed, with cell-cycle regulators (*TYMS*, *CCNB1*, *TTK*) more prominent in mild COVID-19, and genes linked to metabolic adaptation, translation, and cytoskeletal remodeling (*RAC1*, *RPS16*, *IGF1*) emerging in severe disease. These patterns suggest a transition from cell-cycle–dominant programs toward sustained metabolic and structural remodeling under prolonged immune stress [41]. The recurrence of *IGF1* and *MMP9* across both TNBC and non-TNBC breast cancer underscores their central roles at the intersection of oncogenic signaling, immune communication, and COVID-19-associated inflammatory stress.

Across all three cancer types, the consistent identification of hub genes involved in immune signaling (e.g., *IL1B*, *NFKB1*), cell proliferation (e.g., *CCNB1*, *TTK*), and extracellular interactions (e.g., *MMP9*, *PXN)* reinforces the notion that SARS-CoV-2 infection amplifies signaling programs that are already dysregulated within tumor ecosystems. At the same time, the presence of cancer-specific hub genes highlights important disease-context dependencies, suggesting that COVID-19 does not uniformly affect tumor biology but modulates immune–tumor communication differently depending on cancer subtype, baseline immune architecture, and infection severity.

Functional enrichment analysis based on GO and KEGG pathways revealed that COVID-19 shares extensive immunological, oncogenic, and metabolic signaling pathways with TNBC, ccRCC, and breast cancer, with clear differences depending on SARS-CoV-2 infection severity. Rather than reflecting static overlap, these patterns indicate severity-dependent shifts in immune signaling, proliferative stress, and tumor–microenvironment interactions.

In the mild COVID-19–TNBC group, enriched biological processes such as positive regulation of the MAPK cascade, ERK1/ERK2 signaling, cell migration, and angiogenesis reflect early inflammatory and proliferative responses that are characteristic of both TNBC biology and moderate viral immune activation. Molecular function terms, including interleukin-1 receptor binding and IGF receptor binding, point to coordinated cytokine and growth factor signaling, which are known contributors to TNBC aggressiveness and immune modulation during SARS-CoV-2 infection [42]. Enrichment of cellular components such as the extracellular space and plasma membrane further suggests enhanced secretion and receptor-mediated communication, highlighting active immune–tumor and cell–cell signaling. Correspondingly, KEGG pathways including MAPK, Rap1, and Ras signaling—core regulators of survival, proliferation, and inflammation—were dominant [43]. Additional enrichment of steroidogenesis and pathways in cancer may reflect early endocrine-independent adaptation and proliferative signaling under moderate immune stress.

In contrast, severe COVID-19–TNBC showed stronger enrichment of mitotic and proliferative processes, including positive regulation of mitotic nuclear division and cell population proliferation, indicating heightened proliferative pressure under severe immune stress. Concurrent enrichment of cytokine activity and growth factor activity is consistent with immune overactivation and cytokine-driven signaling observed in severe COVID-19, which may exacerbate tumor aggressiveness [44]. KEGG pathways such as PI3K–Akt signaling, focal adhesion, and endocrine resistance—key mediators of cell survival, migration, and therapy resistance—were prominently enriched [45]. When all COVID-19–TNBC samples were analyzed collectively, enrichment patterns reflected a balance between inflammatory signaling and proliferative programs, with cellular components such as extracellular exosomes and extracellular matrix underscoring tumor microenvironment remodeling and intercellular communication relevant to both infection and metastasis.

ccRCC was analyzed within the context of an immune-inflamed yet functionally constrained tumor microenvironment characterized by immune infiltration, immune exhaustion, and dysregulated signaling. In mild COVID-19–ccRCC, GO enrichment was dominated by immune regulation, antiviral defense, and signal transduction processes, suggesting immune activation or dysregulation within the tumor context. KEGG pathways highlighted mTOR and Ras signaling, reflecting metabolic and oncogenic convergence with pathways also observed in breast cancer. In severe COVID-19–ccRCC, enrichment shifted toward processes involving cytoskeletal reorganization, vesicle trafficking, and neurodevelopmental signaling, which may contribute to tumor invasion, angiogenesis, and altered immune–tumor communication. KEGG analysis further emphasized metabolic reprogramming and insulin/IGF-related signaling, consistent with metabolic stress and proliferative adaptation. Across all COVID-19–ccRCC comparisons, dominant GO terms related to cell cycle control, energy metabolism, and signaling, while enriched KEGG pathways involved angiogenesis, vascular regulation, and cell adhesion, aligning with ccRCC progression and vascular remodeling.

In breast cancer, mild COVID-19 was characterized by enrichment of GO terms related to translation, ribosome biogenesis, cytoplasmic translation, and metal ion binding, reflecting increased protein synthesis, metabolic activity, and oxidative stress—features common to both tumor biology and viral infection responses. Corresponding KEGG pathways, including Coronavirus disease–COVID-19, pathways in cancer, Rap1 signaling, and vascular smooth muscle contraction, support functional crosstalk between viral immune responses, vascular changes, and tumor signaling. In severe COVID-19-associated breast cancer, enrichment shifted toward cell adhesion, angiogenesis, plasma membrane organization, and protein binding, suggesting enhanced invasiveness, extracellular matrix remodeling, and angiogenic activity. KEGG pathways such as focal adhesion, cytoskeleton regulation, ribosome, and breast cancer signaling reinforce themes of structural remodeling, proliferation, and sustained metabolic demand. Across all COVID-19–breast cancer samples, enrichment of G protein–coupled receptor signaling, axon extension, and extracellular space indicates the co-option of developmental and morphogenetic pathways that support tumor progression and immune evasion. KEGG pathways including Wnt and cGMP–PKG signaling further highlight the role of developmental signaling and vascular dynamics in aggressive tumor behavior.

Across all cancer types, COVID-19 severity markedly influenced the enrichment of oncogenic, immune, and metabolic pathways, suggesting that severe viral infection may act as a biological amplifier of pre-existing tumor-associated signaling programs. Shared pathways—including cytokine–cytokine receptor interaction, MAPK signaling, proteoglycans in cancer, Ras signaling, and Wnt signaling—underscore their central roles in both viral pathogenesis and cancer progression. Collectively, these findings support a model in which increasing immune stress during severe COVID-19 exacerbates dysregulated immune communication, metabolic reprogramming, and proliferative signaling in aggressive cancers, reinforcing the importance of severity-aware evaluation of virus–cancer interactions and informing future stratified therapeutic strategies.

To gain insight into the regulatory mechanisms governing the expression of shared hub genes among ccRCC, COVID-19, TNBC, and BC, we constructed TF–gene and miRNA–gene interaction networks. These networks highlight putative upstream regulators that may coordinate immune, inflammatory, and stress-response programs common to both viral infection and tumor biology. In COVID-19-TNBC, the strong regulatory role of inflammatory TFs like NFKB1 and RELA reflects the immune-related overlap between TNBC and COVID-19. miRNAs, such as “miR-145-5p”, are known tumor suppressors that can mediate both antiviral and anticancer roles. In COVID-19-ccRCC, “miR-192-5p” and “miR-215-5p” are associated with renal cancer progression and are possibly modulated during viral infection. The presence of BRCA1 and NFKB1 suggests that DNA repair and inflammation share common pathways. In COVID-19 breast cancer, the involvement of DNA repair and cell cycle TFs such as BRCA1 and TP53 suggests the overlap between COVID-19-induced stress and breast cancer tumorigenesis. Shared miRNAs across cancer types likely reflect generalized stress, apoptotic, and immune-regulatory responses rather than disease-specific effects.

Overall, these regulatory network analyses suggest that COVID-19 severity is associated with modulation of gene regulatory programs that overlap with pathways commonly dysregulated in aggressive cancers. Key recurrent elements—such as *IGF1*, *MMP9*, *NOTCH1*, and TFs including NFKB1 and RELA—emerge as central nodes within immune communication, extracellular remodeling, and stress-response networks shared across disease contexts. Importantly, these interactions represent predicted regulatory relationships inferred from curated databases and integrative network analyses. They should therefore be interpreted as hypothesis-generating signals that highlight potential points of molecular convergence, rather than as experimentally validated mechanisms or evidence of direct clinical interaction.

Taken together, our findings support the interpretation that persistent immune communication dysfunction—rather than isolated inflammation or proliferation alone—represents the dominant shared axis linking COVID-19 severity and aggressive cancers. This includes chronic immune activation, sustained antigen presentation, and progressive immune exhaustion occurring within altered intercellular signaling networks. Recent immune communication network analyses in COVID-19 have demonstrated that exhausted memory CD8^+^ T cells remain centrally engaged in MHC class I–mediated signaling despite elevated inflammatory and exhaustion scores, sustaining chronic immune and metabolic pathway activation in severe and prolonged disease states. Importantly, these features closely parallel immune–tumor communication dynamics observed in aggressive cancers, where persistent antigen exposure, metabolic stress, and dysfunctional immune signaling contribute to immune escape and tumor progression [13].

This study relies on in silico integration of heterogeneous transcriptomic datasets from COVID-19 and cancer cohorts. While this enables the identification of shared genes and pathway-level convergence, it does not directly capture immune cell–cell communication, functional immune states, or causal relationships. Therefore, the results should be interpreted as hypothesis-generating rather than definitive. Limited sample sizes, particularly for COVID-19 cohorts, and incomplete clinical metadata restricted robustness and clinical correlation. The predicted gene, transcription factor, and miRNA interactions were not experimentally validated. In addition, only transcriptomic data were analyzed, and immune communication was inferred indirectly through network-based approaches. Finally, COVID-19 datasets were derived from the early pandemic period, prior to the emergence of major viral variants. Disease severity was used as a proxy for immune response differences. Future studies integrating longitudinal, variant-specific, and multi-omics data with experimental validation are needed.

The integrative analysis of expression data, pathway enrichment, and regulatory networks supports the hypothesis that COVID-19 and cancer share familiar immunological and signaling landscapes, particularly during severe infections. Importantly, these insights retain clinical relevance beyond the pandemic, informing our preparedness for future viral outbreaks and guiding the management of high-risk cancer patients who may face similar immunological challenges. Understanding the molecular interplay between infectious diseases and cancer is essential for developing integrated, personalized treatment strategies that address the complexity of comorbid conditions.

## 5. Conclusions

This integrative bioinformatics study identifies shared molecular signatures and regulatory networks linking COVID-19 severity with aggressive cancers, including TNBC, breast cancer, and ccRCC. By focusing on transcriptomic overlap and pathway-level convergence, our findings reveal common inflammatory, immune, and proliferative signaling programs observed across severe viral infection and tumor contexts. Although patient-level clinical comparisons were not feasible due to limited metadata, these results provide a systems-level, hypothesis-generating framework that does not imply causality or clinical outcome effects. Collectively, this work offers a conceptual basis for future mechanistic and experimental studies aimed at understanding immune-related vulnerabilities in cancer during current or future viral outbreaks.

## Figures and Tables

**Table 1:** Top hub genes shared between COVID-19 (by severity) and cancer types.

Cancer Type	COVID-19 Severity	Top Shared Hub Genes
TNBC	Mild	*FLT1*, *IL1A*, *IL1B*, *FGF1*, *IGF1*
	Severe	*MMP9*, *IGF1*, *CD44*, *ESR1*, *NOTCH1*
	All COVID-19	*NOTCH1*, *IGF1*, *IL1B*, *IL1A*, *MMP9*
ccRCC	Mild	*TTK*, *ASPM*, *CDCA3*, *ANLN*, *BARD1*
	Severe	*ASPM*, *CCL5*, *ESR1*, *CCNB1*, *CENPE*
	All COVID-19	*TTK*, *CCNB1*, *ASPM*, *RAD51AP1*, *NCAPG*
BC	Mild	*TYMS*, *ASPM*, *TOP2A*, *CCNB1*, *TTK*
	Severe	*MMP9*, *CCNB1*, *IGF1*, *RPS16*, *RAC1*
	All COVID-19	*TOP2A*, *PXN*, *MMP9*, *SPP1*, *IGF1*

**Table 2:** Functional axes and representative hub genes across TNBC, ccRCC, and breast cancer with biological interpretations.

Functional Axis	TNBC—Representative Hub Genes	ccRCC—Representative Hub Genes	Breast Cancer—Representative Hub Genes	Biological Interpretation
Immune exhaustion and chronic inflammation	*IL1A*, *IL1B*, *NOTCH1*	*CCL5*	*SPP1*	Persistent cytokine and chemokine signaling, macrophage activation, and immune dysfunction associated with chronic inflammatory states and inferred T-cell exhaustion in severe COVID-19 and tumor microenvironments [33,34].
Immune–tumor interaction and extracellular remodeling	*MMP9*, *CD44*, *FLT1*	—	*MMP9*, *PXN*, *RAC1*	Extracellular matrix remodeling, vascular and adhesion signaling, and regulation of immune cell trafficking that shape the tumor–immune interface and influence immune access to tumor sites [35,36].
Metabolic and proliferative stress	—	*ASPM*, *CCNB1*, *TTK*, *RAD51AP1*, *NCAPG*, *CENPE*, *CDCA3*	*TOP2A*, *TYMS*, *CCNB1*, *TTK*, *RPS16*	Elevated biosynthetic demand, DNA replication and repair, and translational activity reflecting metabolic adaptation and proliferative stress that may contribute to immune dysfunction under systemic inflammatory conditions [37].

**Table 3:** Integrated severity-stratified molecular landscape across COVID-19 and cancer types.

COVID-19 Severity	Cancer Type	Dominant Biological Theme	Key Shared Hub Genes	Representative GO Terms	Key KEGG Pathways	Key TFs	Key miRNAs
Mild	TNBC	Early immune activation and inflammatory signaling	*FLT1*, *IL1A*, *IL1B*, *FGF1*, *IGF1*	MAPK/ERK cascade, cytokine-mediated signaling, angiogenesis	MAPK, Ras, Rap1, Pathways in cancer	NFKB1, RELA	miR-145-5p
	BC	Proliferative and metabolic adaptation	*TYMS*, *ASPM*, *TOP2A*, *CCNB1*, *TTK*	Translation, ribosome, oxidative stress	Rap1, COVID-19 pathway, Cancer pathways	TP53, BRCA1	miR-34a-5p
	ccRCC	Cell cycle activation with immune modulation	*TTK*, *ASPM*, *CDCA3*, *ANLN*, *BARD1*	Cell cycle, immune regulation, viral defense	mTOR, Ras signaling	TP53, NFKB1	miR-192-5p
Severe	TNBC	Chronic inflammation and immune exhaustion	*MMP9*, *IGF1*, *CD44*, *ESR1*, *NOTCH1*	Cytokine activity, mitotic division, ECM remodeling	PI3K-Akt, Focal adhesion, Endocrine resistance	NFKB1, RELA, ESR1	miR-145-5p
	BC	Immune-driven invasion and cytoskeletal remodeling	*MMP9*, *CCNB1*, *IGF1*, *RPS16*, *RAC1*	Cell adhesion, angiogenesis, cytoskeleton	Focal adhesion, Ribosome, Breast cancer	NFKB1, TP53	miR-215-5p
	ccRCC	Metabolic stress and proliferative signaling	*ASPM*, *CCL5*, *ESR1*, *CCNB1*, *CENPE*	Vesicle trafficking, cytoskeleton organization	Insulin/IGF, Metabolic pathways	RELA, ESR1	miR-192-5p
All COVID-19	TNBC	Persistent immune–tumor communication	*NOTCH1*, *IGF1*, *IL1B*, *IL1A*, *MMP9*	Cytokine signaling, angiogenesis	MAPK, Steroidogenesis	NFKB1, RELA	miR-145-5p
	BC	ECM remodeling and immune signaling	*TOP2A*, *PXN*, *MMP9*, *SPP1*, *IGF1*	Extracellular space, axon extension	Wnt, cGMP-PKG	TP53, BRCA1	miR-34a-5p
	ccRCC	Proliferation and DNA repair under immune stress	*TTK*, *CCNB1*, *ASPM*, *RAD51AP1*, *NCAPG*	Cell cycle, energy metabolism	Angiogenesis, Cell adhesion	TP53, BRCA1	miR-215-5p

## Data Availability

The data supporting the findings of this study are available from the corresponding author upon reasonable request.

## Supplementary Materials

The following supporting information can be downloaded at https://www.mdpi.com/article/10.3390/genes17020150/s1, Table S1: GO and KEGG Enrichment of Shared DEGs in COVID-19–TNBC by Disease Severity; Table S2: GO and KEGG Enrichment of Shared DEGs in COVID-19–ccRCC by Disease Severity; Table S3: GO and KEGG Enrichment of Shared DEGs in COVID-19–Breast Cancer by Disease Severity; Table S4: miRNA–hub gene interactions in COVID-19–TNBC; Table S5: miRNA–hub gene interactions in COVID-19–ccRCC; Table S6: miRNA–hub gene interactions in COVID-19–Breast Cancer; Table S7: TF–hub gene interactions in COVID-19 and TNBC; Table S8: TF–hub gene interactions in COVID-19 and ccRCC; Table S9: TF–hub gene interactions in COVID-19 and BC.
