# Supplementary material for: Genetically Shared Signatures Between COVID-19 and Cancer Identified Through In Silico Case–Control Analysis
Source: Genes (Basel). 2026 Jan 28;17(2):150. doi: 10.3390/genes17020150 (PMC12940708; doi:10.3390/genes17020150)
Supplement: Supplementary file 1 [file genes-17-00150-s001.zip › Supplementary S7-S9 TFs.pdf]

Supplementary Table S7. TF–hub gene interactions in COVID-19 and TNBC

**COVID-19 group**	**Transcription factor**	**Number of hub genes regulated**
M□ld COVID-19–TNBC	E2F1	2
M□ld COVID-19–TNBC	JUN	2
M□ld COVID-19–TNBC	NFKB1	2
M□ld COVID-19–TNBC	RELA	2
Severe COVID-19–TNBC	HDAC1	3
Severe COVID-19–TNBC	SP1	3
Severe COVID-19–TNBC	EP300	2
Severe COVID-19–TNBC	MTA1	2
Severe COVID-19–TNBC	TWIST1	2
Severe COVID-19–TNBC	IKBKB	2
Severe COVID-19–TNBC	SNAI2	2
Severe COVID-19–TNBC	BRCA2	2
Severe COVID-19–TNBC	NCOR1	2
Severe COVID-19–TNBC	STAT5B	2
Severe COVID-19–TNBC	KLF5	2
Severe COVID-19–TNBC	SIRT1	2
All COVID-19–TNBC	SIRT1	4
All COVID-19–TNBC	JUN	4
All COVID-19–TNBC	NFKB1	4
All COVID-19–TNBC	RELA	4
All COVID-19–TNBC	EP300	2
All COVID-19–TNBC	NFKBIA	2
All COVID-19–TNBC	KLF5	2
All COVID-19–TNBC	FOS	2

Supplementary Table S8. TF–hub gene interactions in COVID-19 and ccRCC

COVID-19 group	Transcription factor	Number of hub genes regulated
Mild COVID-19–ccRCC	FOXJ1	1 (ASPM)
Mild COVID-19–ccRCC	BRCA1	1 (ASPM)
Severe COVID-19–ccRCC	BRCA1	3
Severe COVID-19–ccRCC	SP1	2
Severe COVID-19–ccRCC	TP53	2
Severe COVID-19–ccRCC	IRF1	2
Severe COVID-19–ccRCC	NFKB1	2
Severe COVID-19–ccRCC	RELA	2
All COVID-19–ccRCC	BRCA1	2
All COVID-19–ccRCC	E2F4	2

Supplementary Table S9. TF–hub gene interactions in COVID-19 and BC

COVID-19 group	Transcription factor	Number of hub genes regulated
M□ld COVID-19–BC	E2F1	3
M□ld COVID-19–BC	YBX1	3
M□ld COVID-19–BC	BRCA1	2
M□ld COVID-19–BC	E2F4	2
M□ld COVID-19–BC	TP53	2
M□ld COVID-19–BC	USF1	2
Severe COVID-19–BC	EP300	2
Severe COVID-19–BC	BRCA1	2
Severe COVID-19–BC	IRF1	2
Severe COVID-19–BC	KLF5	2
Severe COVID-19–BC	NFKB1	2
Severe COVID-19–BC	RELA	2
Severe COVID-19–BC	TFAP2A	2
Severe COVID-19–BC	PTTG1	2
All COVID-19–BC	EP300	2
All COVID-19–BC	HDAC1	2
All COVID-19–BC	SP1	2
All COVID-19–BC	CEBPA	2
