# Supplementary material for: Genetically Shared Signatures Between COVID-19 and Cancer Identified Through In Silico Case–Control Analysis
Source: Genes (Basel). 2026 Jan 28;17(2):150. doi: 10.3390/genes17020150 (PMC12940708; doi:10.3390/genes17020150)
Supplement: Supplementary file 1 [file genes-17-00150-s001.zip › Supplementary Table S4-S6_mirnas.pdf]

Supplementary Table S4. miRNA–hub gene interactions in COVID-19–TNBC

COVID-19 group	miRNA	Number of hub genes targeted
Mild COVID-19–TNBC	hsa-miR-199a-3p	3
Mild COVID-19–TNBC	hsa-miR-24-3p	2
Mild COVID-19–TNBC	hsa-miR-150-3p	2
Mild COVID-19–TNBC	hsa-miR-130b-5p	2
Mild COVID-19–TNBC	hsa-miR-4753-3p	2
Mild COVID-19–TNBC	hsa-miR-155-5p	2
Severe COVID-19–TNBC	hsa-miR-145-5p	4
Severe COVID-19–TNBC	hsa-miR-129-5p	3
Severe COVID-19–TNBC	hsa-miR-9-5p	3
Severe COVID-19–TNBC	hsa-miR-204-5p	3
Severe COVID-19–TNBC	hsa-miR-211-5p	3
Severe COVID-19–TNBC	hsa-miR-24-3p	2
Severe COVID-19–TNBC	hsa-let-7e-5p	2
Severe COVID-19–TNBC	hsa-miR-302a-5p	2
Severe COVID-19–TNBC	hsa-miR-19b-3p	2
Severe COVID-19–TNBC	hsa-miR-26a-5p	2
Severe COVID-19–TNBC	hsa-miR-130a-3p	2
Severe COVID-19–TNBC	hsa-miR-130b-3p	2
Severe COVID-19–TNBC	hsa-miR-18b-5p	2
Severe COVID-19–TNBC	hsa-miR-29b-3p	2
Severe COVID-19–TNBC	hsa-miR-302a-3p	2
Severe COVID-19–TNBC	hsa-miR-373-3p	2
Severe COVID-19–TNBC	hsa-miR-520a-3p	2
Severe COVID-19–TNBC	hsa-miR-520c-3p	2
Severe COVID-19–TNBC	hsa-miR-3121-5p	2
Severe COVID-19–TNBC	hsa-miR-181a-5p	2
Severe COVID-19–TNBC	hsa-miR-335-3p	2
Severe COVID-19–TNBC	hsa-miR-199a-3p	2
Severe COVID-19–TNBC	hsa-miR-1-3p	2
Severe COVID-19–TNBC	hsa-miR-149-3p	2
Severe COVID-19–TNBC	hsa-miR-130b-5p	2
Severe COVID-19–TNBC	hsa-miR-4728-5p	2
Severe COVID-19–TNBC	hsa-miR-4753-3p	2
Severe COVID-19–TNBC	hsa-miR-6785-5p	2
Severe COVID-19–TNBC	hsa-miR-6883-5p	2
Severe COVID-19–TNBC	hsa-miR-7106-5p	2
Severe COVID-19–TNBC	hsa-miR-30a-5p	2
Severe COVID-19–TNBC	hsa-miR-34a-5p	2
Severe COVID-19–TNBC	hsa-miR-27b-3p	2
Severe COVID-19–TNBC	hsa-miR-608	2
Severe COVID-19–TNBC	hsa-miR-4793-5p	2
Severe COVID-19–TNBC	hsa-miR-5196-3p	2
Severe COVID-19–TNBC	hsa-miR-145-3p	2
Severe COVID-19–TNBC	hsa-miR-15b-5p	2
Severe COVID-19–TNBC	hsa-miR-143-3p	2
Severe COVID-19–TNBC	hsa-miR-320a	2
Severe COVID-19–TNBC	hsa-miR-942-3p	2

COVID-19 group	miRNA	Number of hub genes targeted
All COVID-19–TNBC	hsa-miR-24-3p	3
All COVID-19–TNBC	hsa-miR-204-5p	3
All COVID-19–TNBC	hsa-miR-129-5p	2
All COVID-19–TNBC	hsa-miR-145-5p	2
All COVID-19–TNBC	hsa-let-7e-5p	2
All COVID-19–TNBC	hsa-miR-302a-5p	2
All COVID-19–TNBC	hsa-miR-335-5p	2
All COVID-19–TNBC	hsa-miR-211-5p	2
All COVID-19–TNBC	hsa-miR-9-5p	2
All COVID-19–TNBC	hsa-miR-145-3p	2
All COVID-19–TNBC	hsa-miR-30a-5p	2
All COVID-19–TNBC	hsa-miR-30c-5p	2
All COVID-19–TNBC	hsa-miR-30d-5p	2
All COVID-19–TNBC	hsa-miR-181a-5p	2
All COVID-19–TNBC	hsa-miR-30b-5p	2
All COVID-19–TNBC	hsa-miR-30e-5p	2
All COVID-19–TNBC	hsa-miR-34a-5p	2
All COVID-19–TNBC	hsa-miR-155-5p	2
All COVID-19–TNBC	hsa-miR-21-5p	2
All COVID-19–TNBC	hsa-miR-149-5p	2

Supplementary Table S5. miRNA–hub gene interactions in COVID-19–ccRCC

COVID-19 group	miRNA	Number of hub genes targeted
Mild COVID-19–ccRCC	hsa-miR-192-5p	4
Mild COVID-19–ccRCC	hsa-miR-215-5p	4
Mild COVID-19–ccRCC	hsa-miR-26b-5p	3
Mild COVID-19–ccRCC	hsa-miR-193b-3p	2
Severe COVID-19–ccRCC	hsa-miR-192-5p	3
Severe COVID-19–ccRCC	hsa-miR-20a-5p	2
Severe COVID-19–ccRCC	hsa-let-7g-3p	2
Severe COVID-19–ccRCC	hsa-miR-590-3p	2
Severe COVID-19–ccRCC	hsa-miR-6512-3p	2
Severe COVID-19–ccRCC	hsa-miR-6720-5p	2
Severe COVID-19–ccRCC	hsa-miR-6849-3p	2
Severe COVID-19–ccRCC	hsa-miR-193b-3p	2
Severe COVID-19–ccRCC	hsa-miR-302a-3p	2
Severe COVID-19–ccRCC	hsa-miR-302b-3p	2
Severe COVID-19–ccRCC	hsa-miR-302c-3p hsa-miR-302d-3p	2
Severe COVID-19–ccRCC	hsa-miR-372-3p	2
Severe COVID-19–ccRCC	hsa-miR-373-3p	2
Severe COVID-19–ccRCC	hsa-miR-20b-5p	2
Severe COVID-19–ccRCC	hsa-miR-520e	2
Severe COVID-19–ccRCC	hsa-miR-520a-3p	2
Severe COVID-19–ccRCC	hsa-miR-520b	2
Severe COVID-19–ccRCC	hsa-miR-520c-3p	2
Severe COVID-19–ccRCC	hsa-miR-520d-3p	2
Severe COVID-19–ccRCC	hsa-miR-302e	2
Severe COVID-19–ccRCC	hsa-miR-215-5p	2
All COVID-19–ccRCC	hsa-miR-193b-3p	2
All COVID-19–ccRCC	hsa-miR-26b-5p	2
All COVID-19–ccRCC	hsa-miR-192-5p	2
All COVID-19–ccRCC	hsa-miR-215-5p	2

Supplementary Table S6. miRNA–hub gene interactions in COVID-19–Breast Cancer

COVID-19 group	miRNA	Number of hub genes targeted
Mild COVID-19–BC	hsa-miR-193b-3p	3
Mild COVID-19–BC	hsa-miR-26b-5p	3
Mild COVID-19–BC	hsa-miR-215-5p	3
Mild COVID-19–BC	hsa-let-7b-5p	2
Mild COVID-19–BC	hsa-miR-203a-3p	2
Mild COVID-19–BC	hsa-miR-524-5p	2
Mild COVID-19–BC	hsa-miR-218-5p	2
Mild COVID-19–BC	hsa-miR-192-5p	2
Severe COVID-19–BC	hsa-miR-483-3p	2
Severe COVID-19–BC	hsa-miR-576-5p	2
Severe COVID-19–BC	hsa-miR-132-3p	2
Severe COVID-19–BC	hsa-miR-650	2
Severe COVID-19–BC	hsa-miR-183-3p	2
Severe COVID-19–BC	hsa-miR-3612	2
Severe COVID-19–BC	hsa-let-7e-5p	2
Severe COVID-19–BC	hsa-miR-302a-5p	2
Severe COVID-19–BC	hsa-miR-24-3p	2
Severe COVID-19–BC	hsa-miR-377-5p	2
Severe COVID-19–BC	hsa-miR-455-3p	2
Severe COVID-19–BC	hsa-miR-6086	2
Severe COVID-19–BC	hsa-miR-6134	2
Severe COVID-19–BC	hsa-miR-6499-3p	2
Severe COVID-19–BC	hsa-miR-6516-5p	2
Severe COVID-19–BC	hsa-miR-320a	2
Severe COVID-19–BC	hsa-miR-542-3p	2
All COVID-19–BC	hsa-miR-335-5p	3
All COVID-19–BC	hsa-miR-145-5p	2
All COVID-19–BC	hsa-let-7e-5p	2
All COVID-19–BC	hsa-miR-302a-5p	2
All COVID-19–BC	hsa-miR-21-5p	2
All COVID-19–BC	hsa-miR-524-5p	2
