# Supplementary material for: Genetically Shared Signatures Between COVID-19 and Cancer Identified Through In Silico Case–Control Analysis
Source: Genes (Basel). 2026 Jan 28;17(2):150. doi: 10.3390/genes17020150 (PMC12940708; doi:10.3390/genes17020150)
Supplement: Supplementary file 1 [file genes-17-00150-s001.zip › Supplementary Tables S1-S3-go and kegg terms.pdf]

Supplementary Table S1. GO and KEGG Enrichment of Shared DEGs in COVID-19–TNBC by Disease Severity

COVID-19 Severity	GO Biological Process (BP)	GO Cellular Component (CC)	GO Molecular Function (MF)	Top Enriched KEGG Pathways
**Mild**	Positive regulation of ERK1/ERK2 cascade; Cell adhesion; Cytokine-mediated signaling	Cytoplasmic vesicle; Plasma membrane	Interleukin-1 receptor binding; Integrin binding; Protein binding	Proteoglycans in cancer; Ras signaling pathway; Axon guidance
**Severe**	Positive regulation of MAPK cascade; Inflammatory response; Mitotic nuclear division	Extracellular region; Plasma membrane	Interleukin-1 receptor binding; Cytokine activity	Endocrine resistance; Breast cancer; Cytokine–cytokine receptor interaction; Cancer-related pathways
**All COVID-19**	Regulation of transcription from RNA polymerase II promoter; Cell proliferation; Signal transduction	Extracellular exosome; Plasma membrane; Extracellular space	Cytokine activity; IGF receptor binding; Protein–protein interaction	Breast cancer; Ovarian steroidogenesis; Endocrine resistance; Proteoglycans in cancer

Supplementary Table S2. GO and KEGG Enrichment of Shared DEGs in COVID-19–ccRCC by Disease Severity

COVID-19 Severity	Dominant Biological Themes (GO BP)	Key Cellular Context (GO CC)	Representative Molecular Functions (GO MF)	Key KEGG Pathways
**Mild**	Mitotic cell cycle; Chromosome segregation; Cell division	Spindle; Nuclear chromatin	ATP-dependent microtubule motor activity; Protein binding	Cell cycle; DNA replication
**Severe**	Mitotic nuclear division; DNA repair; Immune cell chemotaxis	Kinetochore; Chromosome centromeric region	Microtubule binding; Chemokine activity	Cell cycle; Chemokine signaling
**All COVID-19**	Cell cycle regulation; DNA damage response; Chromosome organization	Spindle apparatus; Nucleus	Structural molecule activity; ATP binding	Cell cycle; p53 signaling

Supplementary Table S3. GO and KEGG Enrichment of Shared DEGs in COVID-19–Breast Cancer by Disease Severity

COVID-19 Severity	Dominant Biological Themes (GO BP)	Key Cellular Context (GO CC)	Representative Molecular Functions (GO MF)	Key KEGG Pathways
**Mild**	DNA replication; Cell cycle progression; Nucleotide biosynthesis	Chromosomal region; Replication fork	DNA binding; Thymidylate synthase activity	Cell cycle; DNA replication
**Severe**	Inflammatory response; Cell migration; Actin cytoskeleton organization	Extracellular region; Plasma membrane	Cytokine activity; GTPase activity	Cytokine–cytokine receptor interaction; Focal adhesion
**All COVID-19**	Cell proliferation; Immune signaling; Signal transduction	Extracellular vesicle; Plasma membrane	Growth factor binding; Protein–protein interaction	Cancer pathways; PI3K–Akt signaling
